# Hand to Mouth: Shifting the Bare-Minimum Accessibility Paradigm in XR Through Crip-Hacking and Crip-Aesthetics

**DOI:** 10.1007/s11569-025-00494-9

**Published:** 2026-04-13

**Authors:** Puneet Jain, Christian Bayerlein

**Affiliations:** 1 Zurich University of the Arts, Zurich, Switzerland; 2 Concordia University, Montreal, Canada; 3 Koblenz, Germany

**Keywords:** eXtended Reality, Critical disability studies, Mouth interfaces, Assistive technology, Criptastic hacking, Disability aesthetics

## Abstract

eXtended Reality (XR) technologies often embed ableist design assumptions, privileging hand-based interaction and vision-centric interfaces that presume a normative able-bodied user. As a result, many disabled people—including those with limited mobility or blindness—are excluded from the outset, with accessibility added only as an afterthought. We critique this dynamic through a critical disability studies lens, formulating the notion of a “*bare-minimum accessibility paradigm*”—a tendency to meet only minimal compliance requirements rather than rethinking access as a generative design concern. In response, we propose crip-hacking and crip-aesthetics as transformative frameworks for accessible XR design. Crip-hacking draws on disabled communities’ DIY technology adaptations while crip-aesthetics reimagines disability-centric creativity as a design asset. We illustrate these approaches through an autoethnographic account of an XR artwork co-created with disabled artists using mouth gestures. This case demonstrates how reimagining XR through disability experiences challenges entrenched ableist design norms and broadens the discourse at the nexus of disability theory, technology ethics, and inclusive design research.

## Introduction

*“How many of you in the audience today know friends or loved ones who have hands?”*

Addressing the audience with this rhetorical question at the Oculus Connect^1^ conference in 2019, Robert Wang, the director of Meta Reality Labs (formerly Facebook Reality Labs), drew the intended laughter from the crowd as Meta launched ‘hand tracking’ as a new technical feature in XR. eXtended Reality (or XR) is an umbrella term for immersive Virtual/Augmented (VR/AR) Reality environments, experienced using Meta’s head-mounted devices. These XR devices, with the ‘hand tracking’ add-on, were now equipped with embedded cameras and sophisticated computer-vision algorithms, enabling the devices to track the position of one’s hands, finger movements, and hand gestures. This, in turn, would allow users to interact within virtual environments using their bare hands instead of controllers—a breakthrough Meta described as *hand presence*: “the sensation that your own hands were actually there with you in a virtual environment” [1].

Supported by the audience laughter, Wang framed his remark as humorous and self-evident, expressing that the universality of hands is indeed the primary reason why “hardworking researchers and engineers at Meta have come together to address the important topic of hand tracking” [2]. However, this moment also exposed a deeper issue: the ableist design logic under the guise of inclusion. By presenting hands as universally available and intuitive, this framing implicitly stated the exclusion of certain bodies from using XR—such as Eric Desrosiers and Christian Bayerlein who live with quadriplegia and tetraplegia respectively.

Eric Desrosiers and Christian Bayerlein are disabled artists and activists who live with Muscular Dystrophy and Spinal Muscular Atrophy, respectively—disabilities that significantly limit movement in both the upper and lower parts of their bodies. As a result, neither Eric nor Christian can use their hands or fingers to interact with XR devices. They do not have the manual dexterity required to make Meta’s recommended hand gestures, hold controllers or press keys, making conventional XR interaction paradigms [3] largely inaccessible to them. Such exclusionary nature of these technologies puts into question what Wang’s colleague at TaleSpin (another VR company partnering with Meta), stated in the follow-up presentation after Wang, that their *mission is to engage and empower “everyone”* [2]. Meta in their ‘Hand Tracking Deep Dive’ talk at the conference thus exemplified how as one of the leading producers of XR technologies, they are setting forth a narrative in the planning of the future of ‘Metaverse’. *Metaverse*, the new social media platform, what Meta claims to be the “*next evolution in social connection and the successor to the mobile internet”* accessible using the XR devices, was thus showcased as a privileged space–time designed and developed by the able-bodied—for the able-bodied.

This paper takes Meta’s proclamation at the Oculus Connect conference as a critical departure point to examine how current XR shapes dominant assumptions about how bodies should move and interact in spaces of the Metaverse. These technologies, we argue, reinforce ableist ideologies by encoding normative expectations of vision, movement, and control—thereby establishing what we call an *accessibility paradigm*. Firstly, we examine how the dominance of vision, or ocular-centrism, structures the very ontology of the Metaverse and informs Human–Computer Interaction (HCI) research on assistive XR, particularly in its engagement with blind and low-vision users. Secondly, by drawing on literature from critical disability studies, we argue that HCI’s existing approaches to accessibility remain constrained within, what we term as *a bare-minimum accessibility paradigm*—a framework that treats access as functional compliance rather than as a site of creative, aesthetic, or political inquiry.

Finally, to propose an alternative, this paper offers an auto-ethnographic reflection grounded in the first author (Jain's) artistic collaboration with disabled artists Eric and Christian. Through this collaboration, we trace a shift from hand-centric to mouth-based interaction—not as a technical solution, but as a reorientation of how access, agency, and embodiment in XR might be conceived. By co-creating artistic XR experiences with Eric and Christian, we explore how *crip-hacking* [4] and *crip aesthetics* [5]^2^ can disrupt normative design assumptions in XR and offer a reimagining of “accessibility” through space-body interaction in XR—grounded in the lived experiences and expertise of disabled people. As a conceptual provocation, we ask: *what forms of access become possible when XR is designed from bodies that have never been centered in its imagined user?*

## Positionality and Citational Justice

This paper emerges through long-term collaboration with disabled artists and activists, Eric Desrosiers and Christian Bayerlein. Their lived experiences, artistic practices, and creative engagements with technology are foundational to the arguments presented here. Eric, while central to the development of our ideas and practices, does not engage in academic discourses. Nevertheless, his contributions are acknowledged throughout, and this paper would not exist without his insights and collaborative labor. Christian, in contrast, actively engages with research contexts and is a co-author and co-researcher in this work. We take inspiration from Kumar and Karusala’s call for citational justice in HCI [6], recognizing that authorship and citation are not merely formalities but ethical practices that shape whose knowledge counts. This positionality affirms our commitment to consent, collaboration, and recognition as central to the ethics of disability and technology research.

## Reinforcing Ableism—Ocular-Centrism in XR

Experiencing XR environments requires the augmentation of head-mounted displays (HMDs), hand-held controllers, and body-worn sensor technologies that enable one to merge within a spectrum of physical and virtual space [7]. On one end of the spectrum, XR is termed as VR (or Virtual Reality) which occludes the physical environment and immerses the user in a fully digital 360-degree world. Towards the other end of the spectrum, XR is termed AR (or Augmented Reality) when computer-generated information is overlayed in physical space (e.g. a virtual ball in one’s living room). Between these poles is Mixed Reality (MR), where virtual objects not only coexist with but also respond to the physical environment—for example, a virtual ball that bounces off a real couch. The description of these spaces is essential to understand how the virtual and the physical are arranged in such XR environments – freeing but also constraining the means of interaction, movement, and access.

It is hardly surprising that the most common terminology used to describe the XR headsets is head-mounted “displays” (HMDs). Often seen as a successor to previous screen-based media such as television, phones or computers, these HMDs exemplify the field’s deep-rooted emphasis on vision to access such technologies. Mounting more than half a kilogram device over one’s head; one must block their view of the real-physical space to enter the virtual world—The Metaverse—demanding sight as its ticket of entry. As Jonathan Crary observes, “Modernity is inseparable from the techniques of the observer, and central to those is the regime of vision” [8]. In XR, this regime is amplified—vision is not only the gateway to the virtual world but also the precondition for interaction itself.

However, this prioritization of vision in XR is not merely a technical decision, but has philosophical inheritance, tracing back to Aristotle who claimed sight as the most informative of all the senses [9]. In a similar vein, Descartes asserted “All the management of our lives depends on the senses, and since that of sight is the most comprehensive and the noblest of these, there is no doubt that the inventions which serve to augment its power are among the most useful that there can be” [10], giving sight as the highest priority among other senses. While such ocular-centrism, that is, the dominant ideology that vision is the most important sense over all other senses has been critiqued and challenged by scholars from Media Studies [11], Anthropology [12], Philosophy [13], and Sensory Studies [14], it continues to underpin how access and interaction are imagined in XR environments. As a result, the Metaverse is often implicitly constructed as a space for the “seeing” subject—an assumption that sidelines those with visual disabilities. This raises a critical question: what kinds of bodies are these XR technologies built for, and who remains peripheral to their design?

This vision-centric paradigm extends further into the hardware of the head-mounted displays such as Meta Quest headsets which are mounted with small infrared cameras around the headset for “sensing” the physical environment thus enabling the XR device to do what engineers call “inside-out tracking”. Integration of machine-learning and computer-vision algorithms, the XR headsets (using cameras) generate a real-time 3D map of the immediate surroundings and compute the XR users’ position and coordinates in the space. This mapping enables the device to detect and track the user’s bodily data such as head orientation, hand and finger movements and the patterns in gait in the space. The webpage from Meta Reality Labs, providing the specification of this in-built “SLAM” (or Simultaneous localization and mapping) technology claims that the company “recorded thousands of hours of video in a wide range of sample environments, and then they used the footage to teach the system to identify features in its environment.”

By spotting and tracking, say, the corners of a couch or the edge of a table, Oculus Insight can triangulate a person’s exact location within a room, in real time - similar to the way our eyes detect objects to help orient us [15].

These XR devices proliferated with cameras and sensors hence use vision at their disposal to perform surveillance through machinic gaze, exhausting their dependency on sight to collect bodily-data such as facial expressions, gaps between the fingers, orientation of head, position of hands and movements in 3-D space—reifying the ocular-centrism—extending it into the physical space while our eyes navigate the virtual space. While this system of tracking and spatial computation is celebrated for its precision and immersive potential, it simultaneously raises questions about whose ways of perceiving and navigating the world are privileged—and whose are excluded or overlooked.

Chancey Fleet, a blind writer on assistive technologies for the sight impaired, shares how the absence of the ‘surveillance of sight’ can actually “*make space for other ways of knowing*” [16]. In her post titled ‘*Accessibility, Augmented*’, Fleet describes various technologies that guide and assist her in everyday navigation in the world such as guide dogs, her cane, her friends and various mobile applications. She shares: “Birdsong, low to the ground, implies a hedge; a loose coalition of rolling bags marking the entrance to Penn Station; percussive feet on metal flag the stairs up to the High Line”, all—helping her navigate and become what she likes to call herself, “a *chaotic good traveler*”.

Blindness is, for me, a journey of linear discovery and granular illumination. The absence of a surveying glance makes space for other ways of knowing. Each tap of my cane returns an echo suggesting the dimensions and textures of a space and affords an extended sense of touch that reveals whether my path is flat or terraced, marble or mud [16].

The interdependency of Fleet on humans, animals, and technology as an assemblage to navigate in her daily life reflects how dominance of vision pre-supposes “alienation, solitude, and detachment in the technological world today” [17]. The hegemonic eye as per Architect Juhani Pallasmaa, has a strong tendency to fixate, totalize and thus “seeks domination over all fields of cultural production, and it seems to weaken our capacity for empathy, compassion, and participation with the world” [17]. This persistence of ocular-centrism, from XR’s hardware architecture to its design metaphors and cultural imaginaries, demonstrates how vision is not merely a sensory modality in XR, but a structuring logic. Technologies like inside-out tracking or SLAM do not just “see” for the user; they enforce a mode of interaction that assumes the user can be seen and can see in return.

It is also important to note that while Meta (and other XR enthusiast companies) have increasingly invested in haptics and tactile feedback (e.g. in VR controllers, haptic suits) [18], these developments should not be understood as a challenge to ocular-centrism per se. Rather, haptics in XR are often framed as an additive enhancement—an attempt to integrate more sensory channels into an already vision-dominated interface. In this sense, XR design remains grounded in a normative sensory model that presumes a fully sighted, hand-capable user equipped to seamlessly engage all five [Aristotelian] senses [19]. This raises a critical question: does such an ideology position access in XR as a process of assimilating disabled users into the logic of the able-bodied, sighted norm?

### Continued Ocular-Centrism: From Cultural to Disciplinary Practice

The dominance of vision as a default sensory mode is not only a cultural or philosophical issue—it also finds expression in technical disciplines such as Human–Computer Interaction (HCI), that aim to build accessible XR systems. Recently, HCI expanded its research for making XR accessible for people with disabilities, predominantly focusing on people with blindness and low vision [20–23]. However, ocular-centrism continues to shape even those projects by often reproducing ableist assumptions through exclusionary design processes: insufficient collaboration with disabled people and an over-reliance on visual metaphors and tools. For example, HCI researchers Kremeier and Götzelmann in their 2020 study, tested a VR treadmill to investigate locomotion technique for people with visual impairment in VR [24]. Using a method called ‘walk-in-place,’ their goal was to enable blind users to walk in VR, unconstrained by the limits of physical space, what they described as “immersive travel with less simulator sickness in VR.” The system combined off-the-shelf VR components: a Cyberith Virtualizer treadmill, headphones, and a virtual cane offering vibrotactile feedback. While the study confirms user testing with people with visual impairment, it remains unclear how the participants’ embodied experiences and feedback shaped the design itself. For example, instead of engaging with the real-world practices of white cane usage—an essential tool in blind navigation—the system simulated cane interaction through controller-based vibration.

This abstraction overlooks the cane’s intimate role in blind embodiment, as illustrated by John Hull in *Touching the Rock* [25]. Recounting a dream in which Hull loses his white cane, he writes, “What worried me was not so much how I would get around, but the fact that I had lost a piece of my property”, reflecting that for many blind individuals, the cane is not a disposable aid but a meaningful and embodied practice of navigation, memory, and culture. The cane, he suggests, is not just a tool, but a part of the self—so fundamental that forgetting it feels unthinkable. In contrast, the HCI researchers final claim at the end of the study was that their presented walk-in-place approach (tested using 6 “subjects”) is particularly interesting because one can explore unlimited large environments and use [virtual] white cane exploration as in *“real life”*. Yet, this assumption still invites critical reflection: how is “real life” being interpreted here, and to what extent does the design replicate or diverge from the actual sensory and spatial practices of blind users?

While the assistive prototype was tested by blind participants, it remains unclear whether they were meaningfully involved in the early design and development stages. This temporal gap, where disabled users are brought in only after technical decisions have already been made—reflects a broader pattern in assistive technology design, where disability is approached as a problem to be solved rather than a culture to be engaged with. As a result, essential disability tools like the white cane are generally replaced rather than meaningfully integrated, reinforcing what disability advocate Liz Jackson call a “*disability dongle*”. Jackson created the term “Disability Dongle” [26] to define “a disability aid that is meant to solve a real or perceived problem experienced by disabled people, but which was built without including disabled people in the design process*.*” Blind technologist, Alex Lee, writes “scientists and researchers have tried to re-engineer the cane, attempting to make it better with the use of technology like ultrasonic alerts, vibrations and GPS. But in some ways the humble white cane has proved to be unimprovable” [27]. Hence, amplifying the ocular-centric design principle, the researchers dismiss that white cane users may have knowledge that its designer doesn’t. As Jackson further argues, “This devaluation of existing disabled users is essential to creating a Disability Dongle: it can’t solve a problem disabled people never knew they had”. Mad/crip/queer scholar Adan Jerreat-Poole who lives with chronic pain and uses a cane, similarly, shares how VR limits their interaction with space restricting them to perform and execute “*creative uses of [their] body elbows, armpits, and teeth*” [28]. In real-life one uses feet, knees, hips, elbows, chins, and mouths to push, open, close, throw, and carry objects but the virtual space ignores such possibilities.

Unlike in real life, where I can rest my cane against my body, hold it gently, or tuck it under my armpit, once my grip on the shovel started to lighten, it fell to the ground...Very quickly, my hand and arm muscles became tired from clutching the [VR] controller tightly and holding my extended arm in the air [28].

Alternatively, in another example, HCI researchers Collins et al. [29] built a framework to create inclusive social VR environments for people with blindness and low vision (BLV). Their framework allows a person with BLV to get support from a sighted user to navigate in VR. As the researcher’s state in their study, “a user can virtually hold on to their guide and move with them, while the guide can describe the [virtual] environment”. Enabling a function called ‘S*hared Movement’*, people with blindness and low vision using this framework can enable their sighted guide to direct them and move along with BLV’s virtual avatar. However, instead of physically holding hands with their sighted guide, the blind users “grab the area around a guide’s avatar by holding down the [controller] trigger while standing within one feet of the guide”. The researchers thus omit the consideration of the intimacy [30] between the guide and the blind user who often might hold arms while walking together or the verbal communication that emerges in the pursuit of walking. Interestingly, the researchers didn't reflect on how the description of the sighted person about the Metaverse space re-proliferated the dominance of sight in their conducted experiment. Contrastingly, anthropologist Gili Hammer, who is a sighted woman, drawing on her ethnographic investigations with the blind communities examines “the ways both blindness and sight are represented and experienced within social relations” [31]. In her ethnographic investigation with blind women, Hammer reflects: “*when we walked down the street arm-in-arm, people commonly stared at us, giving me a look of admiration for helping a blind person*” thus describing how walking with her partner (sighted person) and a blind person named Talia reify dominance of sight in a social context.

As we [Hammer, her partner, and Talia (blind)] walked down the crowded street on our way to have lunch...we suddenly noticed that people were looking at us as though I was leading two blind people, one on each arm. I received stares of admiration and empathy. On other occasions, people thought I was blind myself and allowed themselves to stare at me [31].

Hammer’s ethnographic work reveals how practices like walking together—something that may appear mundane—are in fact rich with social meaning, affect, and complex visual politics. These subtleties are precisely what are missing from the functionalist, task-oriented approaches of assistive XR research. When intimacy is replaced with interaction fidelity, or verbal negotiation is flattened into guided description, we see not just a technological substitution but a cultural erasure of disability. Hammer’s reflections invite us to consider how disability is lived and relational—not simply a limitation to be engineered around.

Such ethnographic perspectives complicate the scope of assistive XR research in Human–Computer Interaction (HCI), which often remains bound by what disability scholar Elizabeth Ellcessor has elsewhere called the “*common sense idea of accessibility*” [32]—a logic that reduces accessibility to a checklist or technical fix. Such a framing implicitly aligns with what Alison Kafer [33] critiques as “compulsory able-bodiedness,” where non-disabled body-minds are assumed as the default users of technology. These assumptions manifest most clearly in XR through a persistent ocular-centrism—an uncritical reliance on visual paradigms of access and navigation.

It is this oversimplification and centering of "normative" sensory experience, that produces what we call the *bare-minimum accessibility paradigm*. In the context of XR, this paradigm not only limits the scope of inclusion but also misses the opportunity to imagine radically different modes of inhabiting virtual space and thinking about access in XR. This iteration of accessibility ultimately reaffirms the normative power of sight, even within efforts aimed at inclusion. We argue that this framework risks rendering disability as a condition to be accommodated rather than a standpoint from which to reimagine design and accessibility itself. Hence, it is within this context that we turn to artistic and co-creative XR practices—not to offer solutions to disability, but to foreground experiences, desires, and modes of perception that exceed the logic of “fixing” and instead invite rethinking of ‘access’ in XR.

## Proposal of a Shift Through Crip-Hacking and Crip Aesthetics

In what follows, the first author, Jain, offers an auto-ethnographic reflection to expand on how crip-hacking [4] and crip-aesthetics [5] when applied as a design strategy in an artistic context can offer ways to go beyond the bare-minimum accessibility paradigm and hence, enable us to re-define and re-imagine access through space-body interaction in XR.

“*How do access and aesthetics intersect, collide, and inform one another?*” asks Amanda Cachia in her curated exhibition ‘What can a body do?’ at Haverford College symposium in/visible: disability and the arts [34]. I did not know this question would soon become an important part of my doctoral studies as I (together with a friend) climbed up the ramp leading to the entrance door of disabled artist and painter Eric Desrosier's home in Montreal, Canada. I was meeting Eric to introduce VR to him and carve out the possibilities to make it accessible. Having worked in engineering labs before, meeting a “user” such as Eric, who has muscular dystrophy, at first seemed informal to me. At the entrance, two dogs wearing mouth-muzzles came running to us, barking and driving auditory cues to him and his caretakers that there were visitors at the door. Eric welcomed us as he moved his manually controlled wheelchair towards us, speaking in French to his dogs, letting them understand that we were friends and meant no harm. As he showed his personal space, it was hard to not notice the choreography of his fingers on the joystick attached to his wheelchair – which he used to control and move without external help.

**Fig. 1:**
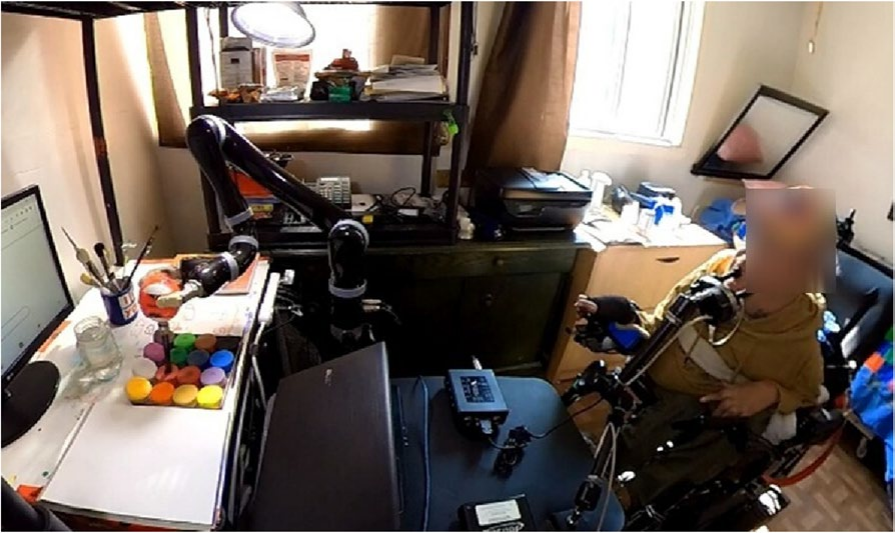
Eric Desrosiers operating a mouth-controlled joystick to paint

The walls of the living room indicated the presence of a painter in the house, as Eric asked us to unhook one of his paintings from the wall so we could see it. Having almost no upper and lower body movements (apart from micro-movements of his fingers to operate his wheelchair), one would wonder, how does he paint? The answer lay in his bedroom where he demonstrated us his skill of operating a newly gifted robotic arm by his friend. The black robotic arm was 3–4 feet tall with three fingers forming a jaw at its very end. Eric, with the help of his caretakers and friends had hacked the device by fixing a rubber ball clinched into ‘the finger-like jaws’ of the robotic arm. Into the rubber ball, he and his caretakers inserted a paintbrush while the wet-colour palette and drawing sheet lay on a table next to the brush. Plugging his mouth-operated joystick (Jouse 3) to his laptop which connects to the robotic arm, Eric demonstrated how he uses his chin, jaw and lip movements to push and rotate the joystick which in turn moves the robotic arm in multiple degrees of freedom. In sequence – by controlling the joystick using his mouth, Eric thus manipulates and moves the jaw of the robotic arm, enabling him to meticulously brush strokes of the paint on the drawing sheet (Fig. 1).

Eric's passion for painting has enabled him to master controlling the scales of movements of the robot arm in space through the micro-gestures of his mouth. With a three-year long practice of using the mouth-operated joystick, an “assistive” technology common to quadriplegic people, Eric has developed unique skills such as dexterity in his mouth movements. In other words, he has substituted the tasks of the two-hands by training his mouth muscles. Exemplifying that “as the affordances offered by physical space shrinked [35]”, Eric hacked and found new affordances in the space through his embodied knowledge of living with a disability. This ability to hack and tinker with space when one is disabled, is what disability scholar Remi M. Yergeau calls ‘criptastic hacking’ [4]. Criptastic hacking is a “methodological approach that harnesses and draws on the long history of hacking and tinkering performed by disabled people to navigate in an inaccessible world” [36]. That is, acknowledgement that disabled people are also hackers and makers who critically tinker with technologies to go “beyond the military-industrial into the realms of activist resistance and world-remaking” [37].

My intention of working with Eric to create accessible XR radically reshaped itself as I found myself in the “aesthetics” of his self-hacked environment. An aesthetic which exemplifies “the process by which human beings attempt to modify themselves, by which they imagine their feelings, forms, and futures in radically different ways” [5]. In other words, an aesthetic that manifests “the human conditions under which it was brought into being” [38]—demonstrating how the lived experiences of disability can inform and make technologies assistive. Disability art-curator Amanda Cachia shares that “Aesthetics opens us to more expansive and diverse conceptions of the human, and disability has become a powerful tool for rethinking human appearance, intelligence, behavior, and creativity” [34]. This was more evident to me as I held Eric's “mouth-painted” painting in my hand which he likes to categorize as abstract art. Just like Meta, I had pre-assumption of *using* micro-movements of Eric's fingers as accessible means of input in VR, that could have enabled him to move and navigate in VR. However, with his painting in my hand, my focus shifted from hand to—the mouth – an unexplored organ (or “input modality” within the discipline of Human–Computer Interaction) to interact in XR. In that moment, Eric was thus no longer a “user” on whom I would test XR but a close collaborator along with whom I would soon hack, tinker and question XR.

### Crip-Hacking

While the common means of interaction in XR are constrained to hand-operated controllers or bare hands (with new hand-tracking functionality of Meta), Eric and I decided to investigate how his existent skill of using his mouth could be used as a means of navigation and interaction in XR. Following the methodology of criptastic hacking (which I will now refer to as ‘crip-hacking’), we first explored computer vision algorithms to track his facial features using a camera. Specifically, we aimed to detect three-dimensional coordinates around the contours of Eric's mouth. Our goal was to determine if, by tracking Eric's face, we could also identify minimal gestures such as the opening of his mouth, by measuring the distance between his upper and lower lips. These movements could then be used to trigger rotation in the virtual environment, allowing Eric to control his point of view in VR using his mouth. Since Eric cannot move his head due to his disability (to have a 360-degree view of the virtual space), this would enable him to navigate the virtual world in a way that suits his abilities.

**Fig. 2:**
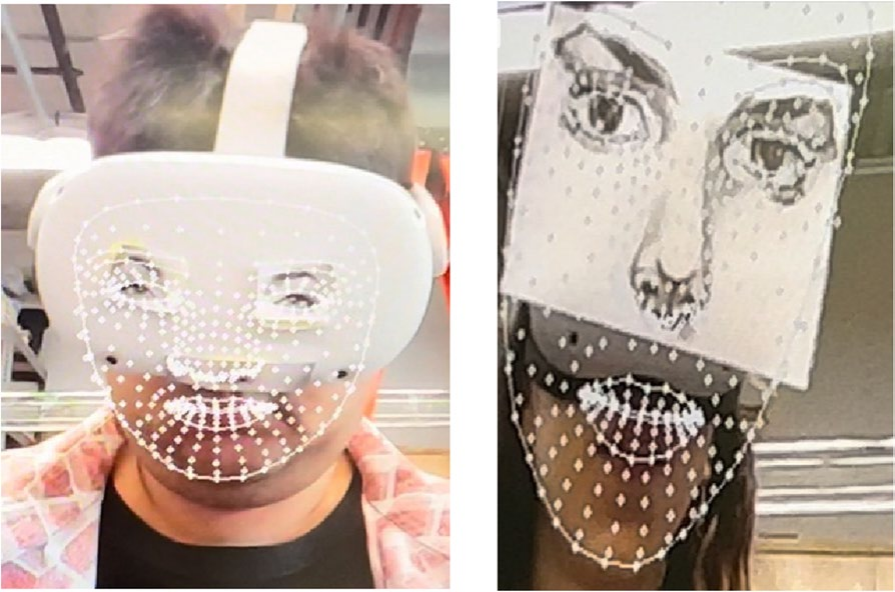
Hacking AI algorithms to track occluded face by pasting paper masks on Meta Quest headsets

However, our labor seemed futile. Since wearing the headset covered Eric's eyes and his upper nose, the algorithm (trained for full face-detection) could not work on occluded faces and hence could not detect his mouth. Eric explained, accessibility is not an end-goal but a process, as we continued exploring creative hacks: such as pasting paper masks of famous celebrities such as Angelina Jolie’s over the headset – tricking the algorithm by juxtaposing Jolie’s upper face and eyes with Eric’ mouth. Voila, it worked! (Fig. 2) But there was a small issue with this hack. Since the tracking of the face requires placement of a stable camera facing Eric, the movement of wheelchair by him or the distance of the camera from his face would disrupt the tracking. We had to find a more robust means of tracking the mouth that is invariant of such movements and is fixed to the headset. We wondered why Meta never thought of such a solution.

We soon discovered that another company (not Meta) investing in XR technologies, HTC, had indeed invested in research on mouth gestures. Yet, such technology did not work the way we intended. HTC, a prime seller of its own XR headsets (the HTC Vive series), had been selling a lip and face tracker (now discontinued) to XR users who use VR chatrooms to meet other people. Since one of the main goals in such VR chat rooms is to meet people and have a conversation, HTC Vive developed this tracker (integrated to the headset) to mimic the facial gestures of the XR users to their virtual avatars’ mouth movements as they talk.

Continuing our hacking process, we thus adopted this tracker to re-modify its software development kit that internally classified 37 types of mouth gestures using machine-learning algorithms (refer to Appendix). We repurposed specific mouth gestures (e.g. moving tongue out) to trigger movements in XR for Eric and later for the second author, Christian Bayerlein, adapting the tracker enabling them to move in VR using mouth movements. As HTC only supports integration with its own headsets, we also engineered a custom solution for Meta Quest compatibility. This involved modifying algorithms, HTC's internal software libraries, and 3D-printing mounts to physically attach the tracker to the headsets.

After multiple trials, we finally came up with a set of six mouth gestures using which the disabled artists Eric and Christian could navigate 360-degree in VR. Gestures such as opening and moving the tongue out, or moving the left chin down opened new ways of interacting with VR space while revealing how disability can teach us to hack, tinker, and modify our interaction with space. Crip-hacking thus opened new epistemic encounters with virtual space – challenging us to re-imagine how disabled bodies could inhabit, alter and access space and time, which Margaret Price has rightly termed as ‘crip space–time’ [39].

### Crip-Aesthetics

While the development of a set of mouth gestures to move and navigate in VR provided Eric and Christian a functional agency to use and experience XR, we extended and integrated this interaction modality to an artwork (showcasing disability culture and living), what we title as *Crip Sensorama*. Crip Sensorama [40, 41], an XR artwork co-developed with Eric Desrosiers and Christian Bayerlein, is a humble nod to Sensorama [42], a physical device from the 1960's that invited the audience to immerse themselves in multisensory stimuli such as stereoscopic colour display, fans, odor-emitters, stereo-sound system, and a motional chair. However, the device, a spectacle of that time which “*simulated immersive environments such as riding a motorcycle through New York”* was clearly not accessible for disabled bodies. Crip Sensorama, therefore, re-imagines this historical much cited example in XR history and reimagines it using the word ‘Crip’ from “Cripple” as a marker of proud and defiant identification of disabled bodies [43] to create another kind of multisensory interactive XR experience that could cast what Tobin Siebers call ‘disability aesthetics’ [5] or what we rephrase here as “crip-aesthetics”. Crip-aesthetics, we frame here as a refusal to hide or minimize the physical realities of disability. Instead, it foregrounds “fragility of the human body” by coupling the body with human–machine interfaces. Hence, turn the very technologies into instruments of critique that have historically marginalized such bodies—to probe “the strong feelings of prejudice that disabled bodies excite in other bodies” [5]. In other words, an aesthetic *that can disrupt the conception of people with disabilities as objects of study but recast disability as something that engenders valuable particularized knowledge.*

The question of rethinking “access” in HCI in this sense, we (I together with Eric and Christian) figured, had to depart from the aesthetics of the mouth—exactly from where it must also be swallowed. The underlying question we asked: Can the mouth help us reimagine “access as an act of perception” [44] by generating “encounters” between the non-disabled and the disabled through the mouth—confronting the many feelings that the non-disabled feel in the presence of the disabled—“fear, pity, fascination, repulsion or merely surprise, none of which is expressible according to social protocol” [45].

**Fig. 3:**
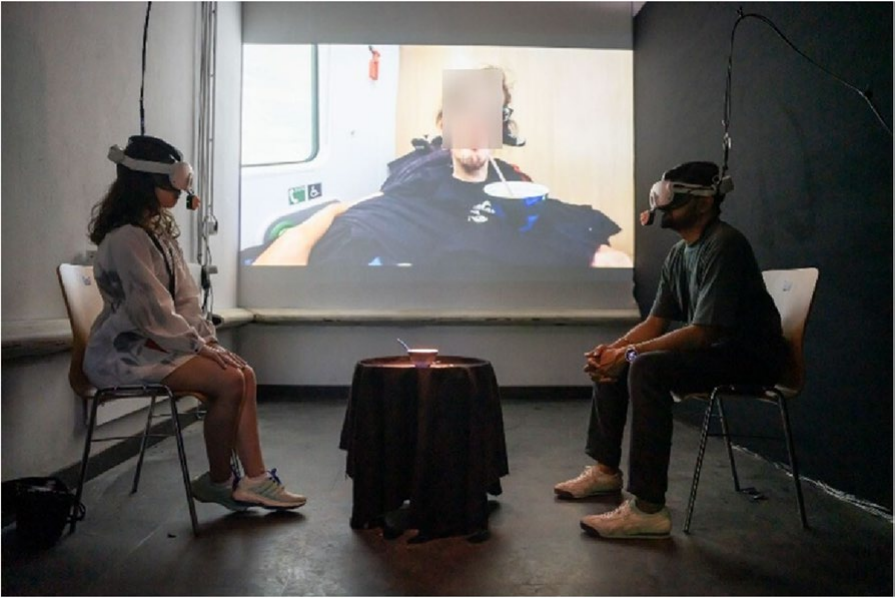
Installation space of *Crip Sensorama*

As a 10–15 min XR experience, in Crip Sensorama, the visitors (in pairs) are invited to enter a dimly lit room, illuminated by the light from a video projection on the wall at the opposite end (Fig. 3). The video shows Christian Bayerlein drinking coffee from his favorite cup using a straw (a daily ritual in his everyday life). Encountering Christian, resting in his wheelchair, slowly drinking his coffee in a fast-paced train (noticeable as one sees rushing landscape through the window beside Christian) the visitors also find themselves in a physical set up consisting of two chairs and a coffee table in between them. The physical set up is arranged so that audience does not face Christian but sit next to him while facing each other. The visitors are then asked to take seats (opposite each other and separated by the coffee table) with Christian's coffee video (no audio) playing beside them on the wall. On the coffee table lies Christian's coffee cup with multiple straws scattered around it. Mounting the XR headsets onto the audience members (integrated with our modified mouth tracker) activates the XR experience, staged in three acts: the extra-ordinary, the ordinary and the infra-ordinary.

**Fig. 4:**
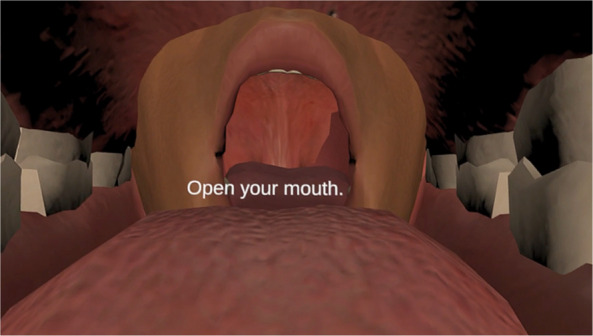
The point of view of a visitor, wearing the XR headset in Crip Sensorama

The extra-ordinary, that is Act 1, situates the audience inside a virtual mouth, with a perspective that they are on a slimy tongue that leads them not to the inside of the body but to another mouth (Fig. 4) – representing the other audience members’ mouth (sitting opposite) but responding to one’s own mouth movements.

**Fig. 5:**
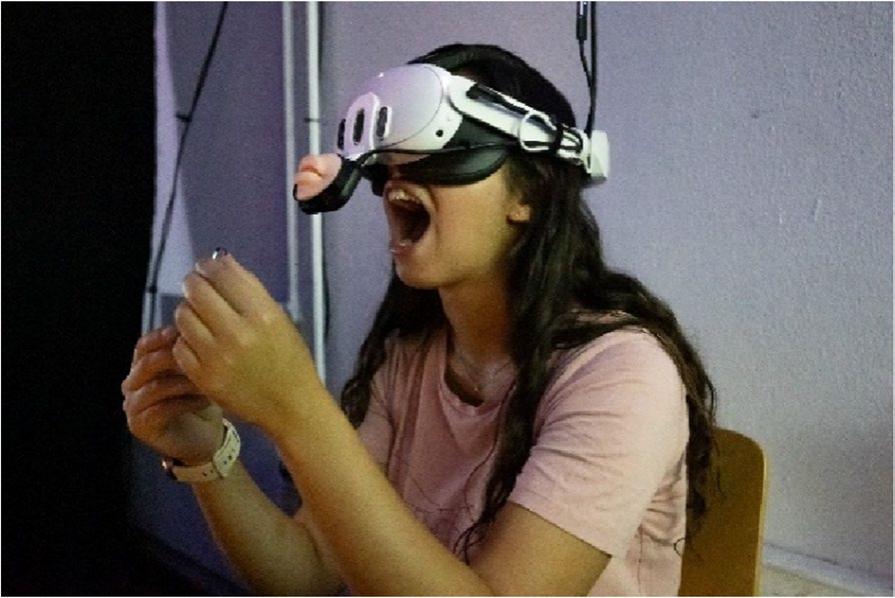
A visitor opening their mouth wide open in while holding Christian's straw in *Crip Sensorama*

Following the prompts which instruct them to open their mouth (Fig. 5), move their lips, tongue, and cheeks, the visitors navigate, move and rotate inside this visceral organ in this first act – encountering the organ which is taken for granted but is a crucial entity in the everyday life of Eric and Christian who use their mouth to paint, use computers and phones, and drive their wheelchairs.

Act 2, *The Ordinary*, prompts both visitors to open their mouths in response to the statement: “Keep your mouth open to encounter the ordinary.” This activates the headset’s *video passthrough*^3^ mode, revealing the physical space (through a live camera feed inside the headset)—a simple, everyday scene. The act of keeping one’s mouth open becomes a way of *staying present*, of maintaining visual access to the world, a sensory task that mirrors Christian's own method of navigating objects and space.^4^ In this moment, the audience witnesses small, habitual gestures—a coffee cup, the act of drinking coffee by Christian on the screen—no longer as trivial but as *meaningful acts of bodily negotiation*. As visitors adjust the mouth gestures, Christian's narration emerges in his own atypical voice—muffled and non-normative (due to his disability) which plays on loop if one closes their mouth, echoing the way many able-bodied people often ask Christian to repeat himself due to difficulties understanding his speech.

It is here, in the seemingly small moment—of adjusting one’s mouth contours to match another’s, of choosing a straw, of hearing a voice on loop—that *Crip Sensorama* invites attention not only to the spectacular or the functional, but to the textures of everyday disabled life. These are the moments that demand slower, more intentional noticing. The final act, *The Infra-ordinary*, draws from French novelist and filmmaker Georges Perec’s idea of the infra-ordinary—not the extraordinary event, but the overlooked detail, the daily habit, the soft repetition of bodily acts we fail to notice [46]. As visitors reach for one of the straws on the table, they are invited to dwell with these objects and gestures differently. What does it mean to drink coffee from a cup without a handle (as Christian doesn’t need one to “hold” onto)—not simply as a sensory experience, but as an act marked by access, by mouth gestures, by dependence or intimacy?

I do not aim to exhaustively describe the artwork, Crip Sensorama, merely through language, but rather to share how the coupling of aesthetics and hacking unfolded and revealed “access” in spaces of XR. In other words, demonstrate how the juxtaposition of crip-aesthetics and crip-hacking to XR technologies can disrupt (if not provoke) normative understanding of bodies by enabling the visitors adjust to other body configurations through machine-body-environment interaction. Henceforth, through active interaction, enabling the audience to speculate new imaginaries of access in XR which offers malleability in space-body engagement and thus opens new affordances for the body when one is “temporarily disabled”. As Eric Desrosiers hacked the spatial arrangements of his home environment enabling him to paint, the audience explored and adapted new hacks such as opening their mouth in XR to observe and hold Christian Bayerlein's coffee cup and his straws (an action they had to perform to unfold the XR experience). While the mouth gestures such as moving the tongue out and opening mouth wide open are not commonly acceptable gestures in public spaces, particularly, in the vicinity of another person, crip-aesthetics and crip-hacking “opened the space” for the same. Might we then ask: what other gestures, bodies, or ways of being have yet to be imagined in XR—not because they are impossible, but because they have been excluded from the outset?

## Conclusion

This paper begins with Meta’s proclamation of the Metaverse to interrogate how contemporary XR technologies are reinforcing ableist design ideologies—particularly through the privileging of vision and manual dexterity as default modes of interaction. We show, how such assumptions are not limited to commercial XR products but extend deeply into accessibility research on XR in HCI, where design solutions for blind users and persons with sensorimotor disabilities often remain tethered to ocular-centric and "normative" frameworks. This convergence results in what we describe as a *bare-minimum accessibility paradigm*—a model that reduces access to functional compliance rather than a site for political, critical, and aesthetic inquiry.

In response, we turn to crip-hacking and crip-aesthetics as generative strategies for rethinking XR from disabled embodiment. The move from hand-centric interaction toward custom mouth-based navigation in XR is not proposed as a better interface, but as a different starting point—one that resists exclusion from the outset by engaging crip expertise as a source of design knowledge. This shift asks not only how disabled users might access XR, but what XR might become when its aesthetics and interaction logics are reimagined through disability.

Looking ahead, this work invites future XR research to move beyond retrofitting access and toward disability-led design practices that open up different ways of thinking about accessibility—beyond compliance—such as through artistic, speculative, or provocative engagements with XR. More broadly, it calls for an ethics of XR that understands access as an ongoing, creative, and accountable practice—one that is shaped *with*, rather than merely for, disabled lives.

## Figures and Tables

**Fig. 6:**
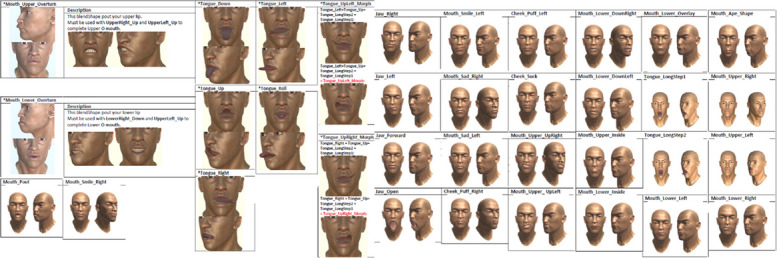
Set of mouth gestures detected by HTC VIVE lip and face tracker

## Data Availability

Not Applicable.

## Footnotes

1 Connect is an annual developer conference organzied by Meta (formerly Facebook) to showcase their research and new innovations in Virtual/Mixed Reality systems.

2 We use “crip-hacking” as shorthand for Yergeau’s “criptastic hacking” [5], referring to disabled practices of tinkering and adaptation. Crip-aesthetics is our extension of Siebers’ “disability aesthetics” [6], to signal aesthetics rooted in lived disability experience. Hence, crip-hacking denotes method, crip-aesthetics denotes artistic expression, and disability aesthetics denotes the broader theoretical framework.

3 Passthrough is a technology in Meta Quest headsets which enables one to see the physical space through a live camera feed inside the headset. This is indeed what allows these headsets to be all: Virtual, Mixed, and Augmented Reality headsets.

4 Christian, like Eric, uses a mouth controlled joystick to operate his wheelchair and travel.
